# Ego-Tucking: a novel psychological mechanism of strategic retrenchment and resilience among Chinese youth

**DOI:** 10.3389/fpsyg.2026.1876249

**Published:** 2026-07-31

**Authors:** Linjun Liu, Yantong Sun, Zongdi Zhao, Luhao Chai, Tianying Xue, Sheng Yan

**Affiliations:** 1 Department of Physical and Aesthetic Education, Wuhan Technical University, Wuhan, China; 2 College of Sports Medicine, Wuhan Sports University, Wuhan, China; 3 Faculty of Pharmacy, Lincoln University College, Selangor, Malaysia; 4 Institute of Sports Technologies and Physical Education, Moscow State University of Sport and Tourism, Moscow, Russia; 5 School of Foreign Economics and Trade, Wuhan Textile University, Wuhan, China; 6 International Cultural Education College, Northeast Agricultural University, Harbin, China

**Keywords:** social psychology, Chinese culture, family education, youth studies, behavioral dynamics

## Abstract

Against the backdrop of intensifying socio-structural pressures and the pervasive “Fo-xi” (Buddha-like) subculture in contemporary China, this study conceptualizes a novel psychological mechanism termed “Ego-Tucking.” Operationalized as a strategic and adaptive defense mechanism, Ego-Tucking denotes an intentional modulation of the self where individuals “tuck away” excessive emotional investment in macro-social competition to preserve psychological equilibrium. Utilizing a multi-dimensional empirical approach with a cohort of university students (*N* = 678), the research identifies a phenomenon of “Functional Bifurcation”: while individuals tactically retreat from high-stakes social hierarchies characterized by “Evaluative Singularity,” they simultaneously maintain high agentic vitality within individualized micro-domains. Path analysis reveals that Internal Psychological Retrenchment (IPR) blue serves as a pivotal mediating bridge, linking external stressors with a state of “Tactical Readiness.” Furthermore, the study highlights how the “Socio-familial Cushion”—rooted in traditional Chinese parental support—provides the material prerequisite for this strategic withdrawal. Ultimately, the findings suggest that Ego-Tucking appears conceptually distinct from learned helplessness or systemic collapse, functioning instead as a form of “Psychological Hibernation” that ensures existential security and psychological resilience in an era of diminishing marginal returns on effort.

## 1 Introduction

In contemporary China, the term “Fo-xi” has emerged as a pervasive sociopsychological construct, denoting a deliberate lifestyle philosophy characterized by emotional detachment and an intentional downregulation of goal-oriented exertion (Shu, 2021; Cui et al., 2024). Unlike generalized youth apathy in other global contexts, “Fo-xi” functions as a pragmatic strategy for emotional self-preservation, deeply rooted in the unique interplay between traditional East Asian equanimity and the modern intensity of the Chinese developmental model. In the present study, we operationalize this phenomenon as Ego-Tucking to achieve conceptual precision. This term captures a dynamic process of intentional ego modulation: individuals “tuck away” excessive self-investment to maintain psychological equilibrium without completely severing their connection to goal-directed activity.

Despite its prevalence, existing psychological frameworks often overlook the internal complexity of this state (Qi and Han, 2025; Yan et al., 2024). While traditional models conceptualize behavioral retreat through a linear paradigm of amotivation, functional deficit, or dynamic adjustment to immediate stress, they fail to account for the unique duality of Ego-Tucking: it is neither a total loss of agency characteristic of learned helplessness nor a flexible coping strategy operating within conventional goal hierarchies. Instead, current theories overlook how individuals enact a simultaneous tactical retrenchment from macro-social competition alongside an active, localized preservation of vitality within individualized micro-domains (i.e., Functional Bifurcation). By introducing Ego-Tucking, this study uniquely fills a theoretical void by conceptualizing a calibrated, self-insulating mechanism of active preservation rather than passive psychological decay.

To address this gap, the present study employs a multi-dimensional approach to empirically validate the mechanism of Ego-Tucking among a cohort of university students (*N* = 678). The investigation focuses on the interplay between distal structural precursors and proximal adaptive responses, specifically examining the relationship between external drivers and the resulting internal psychological retreat. By analyzing the distributional characteristics of these dimensions, we aim to demonstrate that Ego-Tucking represents a state of tactical readiness. This research seeks to provide robust empirical evidence that the strategic containment of the ego serves as a protective insulation, allowing for the re-establishment of meaning and agency within personal micro-domains and ensuring psychological resilience against monolithic social evaluations.

## 2 Results

### 2.1 The structural framework of Ego-Tucking

**Figure 1:**
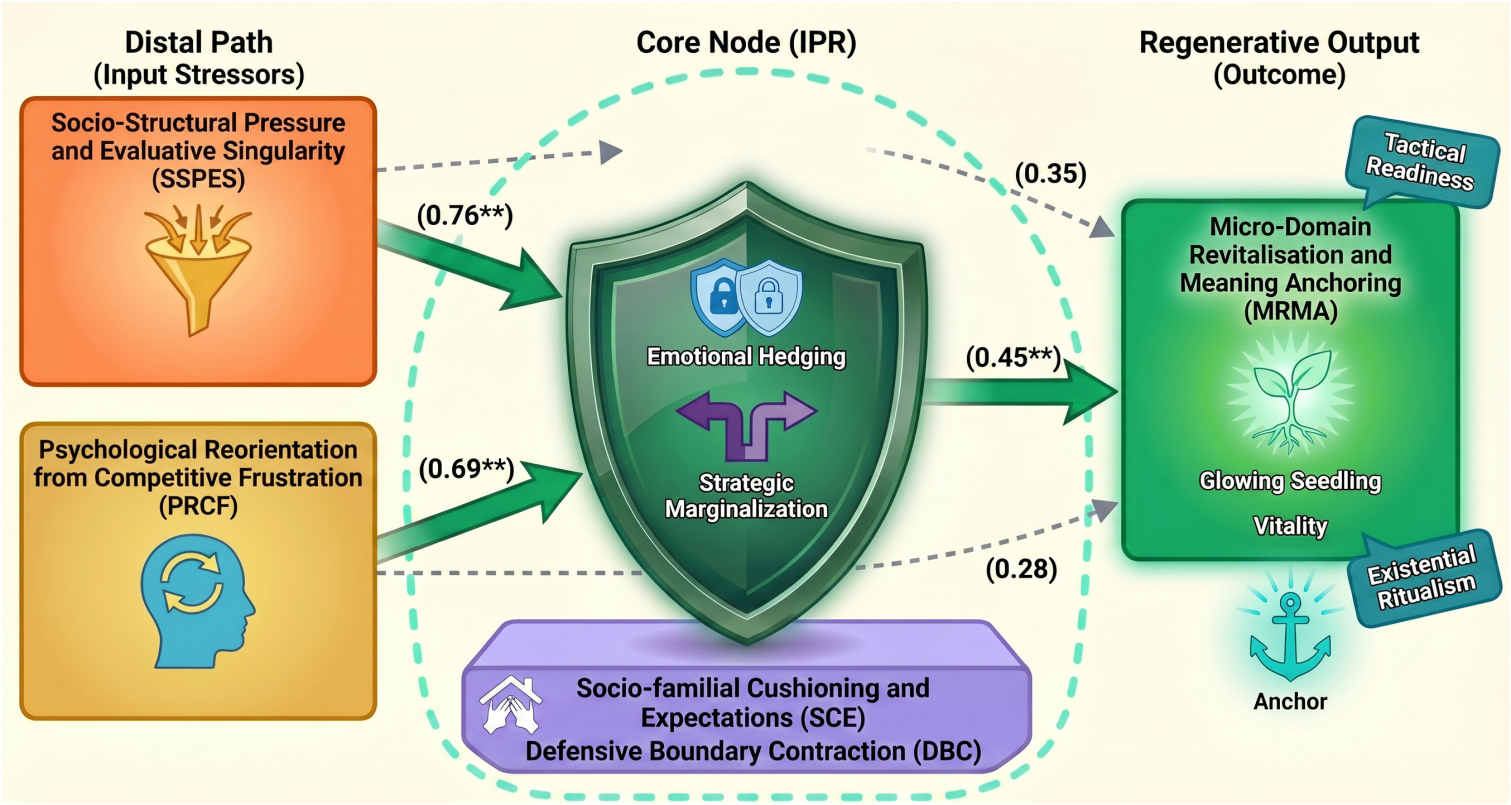
The theoretical framework of strategic retrenchment and meaning anchoring.

To provide a clear overview of the findings, Figure 1 illustrates the mechanistic pathway of “Ego-Tucking” derived from our empirical analysis. The diagram delineates the progression from external stressors—specifically SSPES and PRCF—to the core psychological response of Internal Psychological Retrenchment (IPR). This process is framed by the protective boundary of DBC and supported by SCE, eventually leading to the adaptive outcome of Micro-Domain Revitalization (MRMA). This visual summary serves as the foundation for the detailed statistical results presented in the following sections.

### 2.2 Descriptive statistics of research variables

A descriptive statistical analysis was conducted on the raw total scores of 678 participants to examine the distributional characteristics and data quality of th e measured variables (as shown in Table 1). The results indicated that the mean score for Psychological Reorientation from Competitive Frustration (PRCF; range: 15–75) was 44.70 (*SD* = 9.81). For indicators with a score range of 12–60, Socio-Structural Pressure and Evaluative Singularity (SSPES) and Socio-familial Cushioning and Expectations (SCE) yielded mean scores of 36.01 (*SD* = 8.77) and 31.03 (*SD* = 8.05), respectively. For dimensions ranging from 9 to 45, Micro-Domain Revitalization and Meaning Anchoring (MRMA) exhibited a mean of 31.48 (*SD* = 6.19), and Internal Psychological Retrenchment (IPR) showed a mean of 27.81 (*SD* = 6.86), whereas Defensive Boundary Contraction (DBC; range: 3–15) had a mean of 9.94 (*SD* = 2.77). Statistical indices revealed that the absolute values of skewness (< 0.32) and kurtosis (< 0.50) for all variables remained well within the conventional thresholds for distributional symmetry and stability. These morphological characteristics—specifically the distributional symmetry and stability—confirm that the data is well-behaved and suitable for parametric variance differentiation. Building on this foundational stability, we implement Kelley's (1939) 27% extreme grouping method not merely as a traditional item discrimination check, but as a strategic analytical lens to test our theoretical framework. Specifically, contrasting the extreme tiers of input stressors (SSPES and PRCF) against recovery outputs (MRMA) allows us to empirically verify whether high structural pressure cleanly triggers the “Functional Bifurcation” tipping point, thereby validating the polarized boundaries of the “Ego-Tucking” mechanism without relying solely on linear variance. While path analysis and Pearson correlations establish linear associations across the entire continuous sample, they inherently smooth out localized distributional anomalies and categorical polarization. The extreme-group analysis thus serves as a complementary, necessary diagnostic probe: it uncovers non-linear thresholds and confirms whether the upper-tier cohorts experiencing severe socio-structural pressure actually maintain the distinct statistical pulse of micro-domain revitalization (MRMA) rather than succumbing to a uniform systemic drop in agency.

**Table 1:** Descriptive statistics of the psychological indicators for the study participants.

Variable	Minimum	Maximum	Mean	SD
PRCF	15	75	44.70	9.81
SCE	12	58	31.03	8.05
SSPES	12	60	36.01	8.77
DBC	3	15	9.94	2.77
IPR	9	45	27.81	6.86
MRMA	9	45	31.48	6.19

*n* = 678. The total score ranges for the indicators are: PRCF (15–75), SCE (12–60), SSPES (12–60), DBC (3–15), IPR (9–45), and MRMA (9–45).

### 2.3 Extreme group analysis

The theoretical rationale for Extreme Group Design (EGD) is rooted in the work of Kelley (1939), who demonstrated that utilizing the upper and lower 27% of a distribution provides the optimal balance between maximizing group differentiation and maintaining sufficient sample size for statistical reliability (Preacher et al., 2005; Kelley, 1939). In this study, participants were categorized into the Lower (27%) group and the Upper (27%) group based on their total scores. This 27% ratio is widely recognized as the most efficient threshold for validating the discrimination index, as it maximizes the power to detect significant differences between high and low scorers (Preacher, 2015).

As shown in Table 2, the score thresholds and sample distributions across the six dimensions are presented. For Psychological Reorientation from Competitive Frustration (PRCF), the lower group (scores 15–38) includes 170 participants (25.07%), while the upper group (scores 52–75) consists of 160 participants (23.60%). For Socio-Structural Pressure and Evaluative Singularity (SSPES), the lower group (scores 12–31) comprises 169 participants (24.93%), and the upper group (scores 42–60) includes 163 participants (24.04%).

**Table 2:** Extreme grouping results of research indicators.

Indicator	Group	Score range	Sample size (*n*)	Cumulative percentage (%)
PRCF	Lower	15–38	170	25.07
	Upper	52–75	160	23.60
SCE	Lower	12–25	173	25.52
	Upper	37–60	155	22.86
SSPES	Lower	12–31	169	24.93
	Upper	42–60	163	24.04
DBC	Lower	3–8	163	24.04
	Upper	13–15	107	15.78
IPR	Lower	9–24	151	23.75
	Upper	32–45	178	26.25
MRMA	Lower	9–26	77	11.36
	Upper	36–45	175	25.81

In terms of the behavioral and recovery dimensions, Defensive Boundary Contraction (DBC) shows 163 participants (24.04%) in the lower group (scores 3–8) and 107 participants (15.78%) in the upper group (scores 13–15). Micro-Domain Revitalization and Meaning Anchoring (MRMA) exhibits a lower group (scores 9–26) of 77 participants (11.36%) and an upper group (scores 36–45) of 175 participants (25.81%). Finally, for the core phenomenon of Internal Psychological Retrenchment (IPR), the lower group (scores 9–24) contains 151 participants (23.75%), while the upper group (scores 32–45) consists of 178 participants (26.25%). Additionally, Socio-familial Cushioning and Expectations (SCE) includes 173 participants (25.52%) in the lower group (scores 12–25) and 155 participants (22.86%) in the upper group (scores 37–60).

#### 2.3.1 High-intensity strategic retrenchment without systemic collapse

The extreme group analysis reveals that 26.25% of the participants (*n* = 178) fall into the Upper Group of Internal Psychological Retrenchment (IPR), with scores ranging from 32 to 45. When contrasted with the Competitive Frustration (PRCF) data—where the Upper Group (scores 52–75) comprises 23.60% of the sample–the proportional alignment demonstrates a parallel distributional frequency between external stress metrics and internal reaction tiers.

From an interpretive standpoint, this morphological alignment contrasts with the erratic distribution patterns typically observed in clinical maladaptation. It suggests that internal retrenchment operates in a synchronized manner with external frustration, supporting the theoretical proposition that Ego-Tucking functions as a calibrated internal buffer rather than an indicator of total psychological breakdown.

#### 2.3.2 The “functional bifurcation” paradox: preserved agency in micro-domains

**Figure 2:**
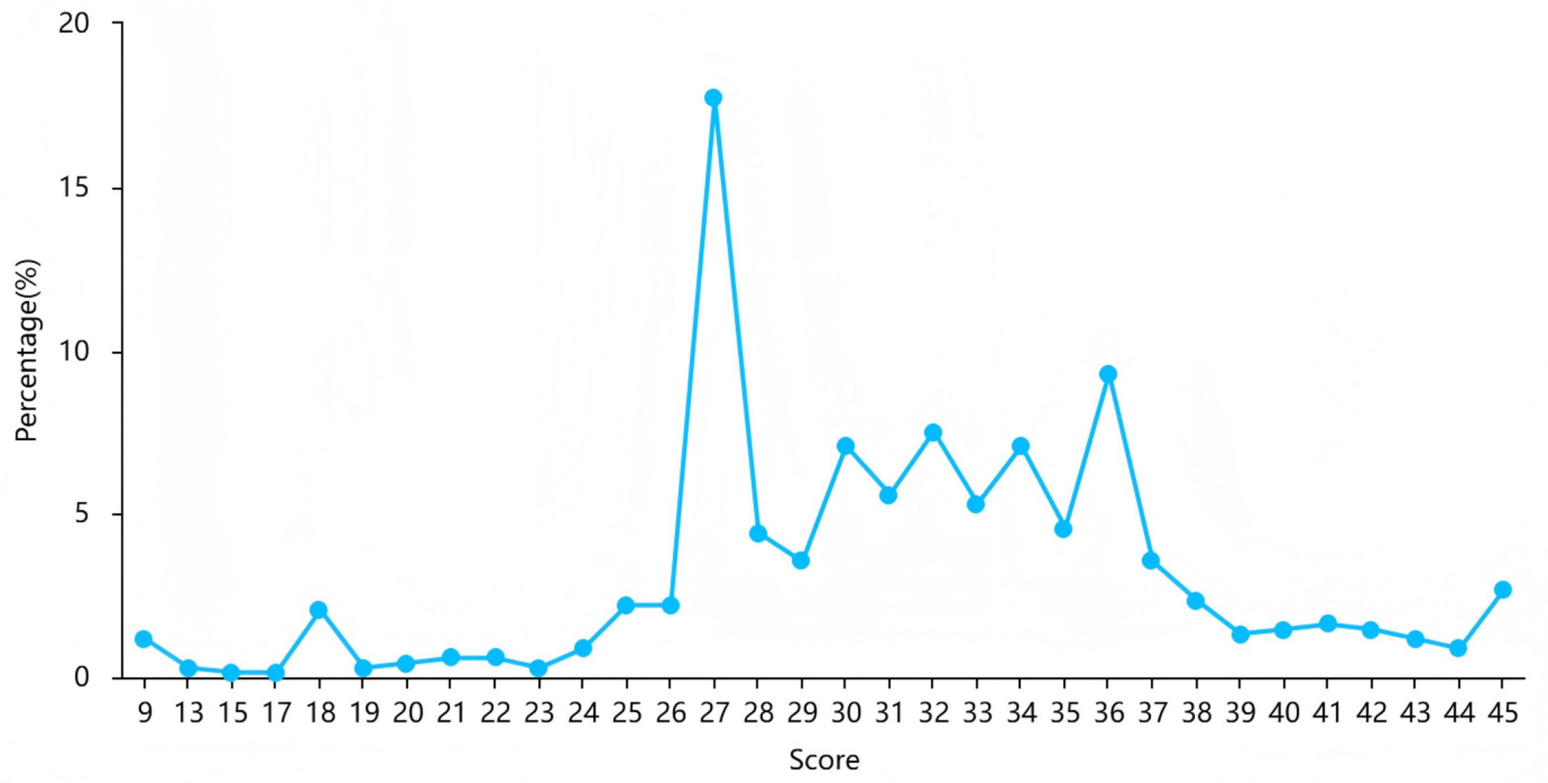
Density and frequency profile of the MRMA.

The frequency distribution of the Micro-Domain Revitalization (MRMA) dimension, as illustrated in Figure 2, demonstrates a prominent empirical concentration occurring at Score 27 (Zhang and Liu, 2025; Yi et al., 2026).

At the theoretical midpoint of the Micro-Domain RevitaliZation (MRMA) dimension, a significant data concentration of 17.70% ($n = 120$) of the participants was observed. Furthermore, the distribution exhibits a high-tier concentration: the Upper Group (scores 36–45), representing 25.81% ($*n* = 175$) of the sample, is more than double the size of the Lower Group (scores 9–26, accounting for 11.36%).

While these localized clustering patterns require longitudinal validation, the persistent mid-to-high score density serves as an empirical baseline indicating that students sustain active response potentials within personal micro-domains. Statistically, this pattern supports the theoretical concept of “Functional Bifurcation,” implying that reduced engagement in macro-hierarchies corresponds with a targeted preservation of localized agency rather than a generalized loss of competence.

The clustering around the mid-to-high score range (Score 27–35), which encompasses 62.83% of the total population, signifies that for the vast majority of “Ego-Tucking” individuals, the private sphere serves as a “Meaning Stronghold.” By preserving such a high level of micro-agency, individuals remain in a state of Tactical Readiness, ensuring they are psychologically prepared for future re-mobilization when external conditions improve.

#### 2.3.3 Calibrated boundaries: distinguishing tactical withdrawal from social isolation

The data regarding Defensive Boundary Contraction (DBC) further elucidates the “strategic” nature of the phenomenon. The Upper Group for DBC represents only 15.78% (Score 13–15), which is significantly lower than the high-score proportions of IPR and MRMA. This discrepancy suggests that even when individuals engage in deep internal retrenchment, they maintain a degree of restraint regarding social isolation. Instead of severing all social ties—a hallmark of pathological withdrawal—the “Ego-Tucking” individual opts for a “Moderate Boundary,” likely supported by the SCE (Socio-familial Cushion), where the Upper Group accounts for 22.86%. This distribution characterizes Ego-Tucking as a state of “Tactical Readiness”; the individual constructs a protective shell to ensure existential security while preserving the potential for rapid re-mobilization if environmental conditions improve.

### 2.4 Distinguishing “Ego-Tucking” from conventional psychological maladaptation

#### 2.4.1 The cultural and structural logic of “Ego-Tucking”

The phenomenon of Ego-Tucking and the resilience demonstrated through Micro-Domain Revitalization (MRMA) are deeply rooted in the specific institutional structures and cultural paradigms of contemporary Chinese society. To understand why this state represents a “strategic hibernation” rather than a permanent loss of agency, one must analyze the synergy between macro-structural constraints and the unique support systems within the Chinese family (Zhang, 2019; Xu et al., 2007; Wong et al., 2006).

Firstly, the Chinese labor market is characterized by a profound “Institutional Singularity.” Social mobility and existential security are increasingly concentrated in a limited number of permanent, tenured positions within the public sector—encompassing the civil service, state-owned enterprises, and public institutions (Ang, 2012; Brødsgaard and Gang, 2014; Burns, 2007). In an era of zero-sum hyper-competition and economic volatility, these positions function as a “Social Safety Shield,” offering a level of stability and welfare that is virtually non-existent in the private market. When individuals perceive that the returns on effort in high-risk sectors have reached a point of exhaustion, Ego-Tucking emerges as a rational “Risk-Averse Social Contract.” By strategically contracting their social ego and professional ambitions, individuals seek long-term systemic refuge within these institutional “shelters,” prioritizing survival over high-stakes, diminishing-return competition.

Secondly, the logic of Ego-Tucking is fundamentally sustained by the traditional Chinese cultural model of parental investment (Sun and Mulvaney, 2022; Wang and Chen, 2024). Unlike the Western paradigm of early individual independence, Chinese parents often provide “Full Life-Cycle Support” to their children. This comprehensive safety net extends far beyond educational tuition to include housing subsidies, the leverage of professional social networks, and long-term subsistence funding. As evidenced by the SCE (Socio-familial Cushioning and Expectations) data, this robust “Socio-familial Cushion” provides the material prerequisite for strategic withdrawal. It allows university students to “opt out” of low-quality, exploitative competition without facing the immediate threat of destitution.

However, this cultural support system operates as a “Double-Edged Sword.” The extensive resources invested by the family carry heavy intergenerational expectations for high-status success and social prestige (Peng et al., 2024; Pan et al., 2021). When individuals find themselves unable to meet these monolithic standards of excellence due to structural rigidity, Ego-Tucking becomes a vital defensive posture. By utilizing the private space afforded by their parents' support, individuals can reorient their focus toward micro-domains (e.g., personal interests, digital subcultures, or private rituals). This allows them to preserve their psychological integrity and “filial dignity” by creating a buffer between intense parental pressure and the harsh realities of the external labor market. Thus, the Chinese family serves as both the architect of the sanctuary and the source of the pressure that necessitates this strategic retreat.

#### 2.4.2 Psychometric differentiation through item design

To establish the theoretical independence of Ego-Tucking, it is essential to analyze the specific logic embedded within the survey instruments. The phrasing of the items, particularly within the PRCF and MRMA scales, reveals that “Ego-Tucking” is an intentional and domain-specific reallocation of psychological resources, rather than a generalized failure of adaptation.

Traditional “learned helplessness” is characterized by a global loss of agency (Tafet and Ortiz Alonso, 2025; Xue et al., 2023). However, the PRCF scale items specifically isolate social competition as the source of exhaustion (e.g., “My current indifferent attitude is specifically targeted at social competition; I believe my competence and talents remain intact within my chosen domains of interest”). This item-level design allows us to distinguish Ego-Tucking from clinical depression or helplessness; the individual does not perceive a total loss of ability, but rather a “Strategic Disengagement” from a specific, zero-sum socio-economic environment. The self-worth remains “tucked” and protected, rather than destroyed.

While “passive avoidance” or “social withdrawal” (e.g., hikikomori) often imply a total severance of meaning-making, the MRMA scale captures a proactive reconstruction of existence (Nonaka et al., 2022). Items such as “Organizing the fragments of daily life with a sense of ritual” and “Establishing self-defined evaluation standards” target what we define as Micro-Agency. From a psychometric standpoint, a high score on these items indicates that the individual is not in a state of apathy. Instead, they are practicing “Existential Hedging”—investing energy into highly controllable micro-domains to buffer against macro-structural turbulence. This proves that Ego-Tucking is a state of “Active Preservation” rather than passive decay.

A hallmark of maladaptive states is the permanent extinction of motivation. In contrast, the MRMA items focusing on “Instantaneous Revitalization” (e.g., “I can instantaneously regain my former drive when presented with an authentic and viable opportunity”) function as a diagnostic filter. This specific question distinguishes the Ego-Tucking state as a form of “Psychological Hibernation” or “Tactical Readiness.” It measures “potential energy” rather than “kinetic energy.” The data confirms that the individual's executive functions are not impaired but are in a “power-saving mode,” awaiting a structural environment that offers a more equitable effort-to-reward ratio.

In conclusion, the survey design demonstrates that Ego-Tucking is an adaptive, culturally-mediated “Protective Shell.” It is a survival strategy that utilizes the “Socio-familial Cushion” of the Chinese family to decouple self-esteem from an exhausted macro-social hierarchy, maintaining the individual's functional integrity for future mobilization.

#### 2.4.3 Quantitative differentiation via data distribution

The distinctiveness of Ego-Tucking as an adaptive survival strategy is empirically supported by the frequency distribution of the Internal Psychological Retrenchment (IPR) and Micro-Domain Revitalization (MRMA) data. By analyzing the concentration of these data points, we can identify a “Strategic Equilibrium” that is statistically distinct from the pervasive deficits typically seen in clinical maladaptation.

In traditional cases of clinical depression or amotivation, data typically exhibit a linear escalation toward the extreme end of the scale (McIntyre et al., 2024; Park et al., 2025). However, the IPR frequency distribution reveals a different pattern: while Scores span from 9 to 45, the vast majority of participants are clustered in the mid-to-high range, specifically between Score 27 (19.62%) and Score 35 (4.57%).

This concentration suggests a “Stabilized Withdrawal.” Unlike the erratic or bottom-heavy distribution of pathological states, the high frequency at the 27-point mark (*n* = 133) indicates that individuals are not experiencing a total collapse of the self. Instead, they are actively maintaining a controlled level of emotional neutrality. They have “tucked” their ego to a specific depth—enough to mitigate the pain of social frustration, but not so deep as to sever their connection with reality or personal agency.

The statistical signature of Ego-Tucking is characterized by a “Non-Zero Baseline” of psychological activity. Despite the high retrenchment Scores in the macro-social sphere (as shown by the cumulative 80.38% of the sample scoring above the median in IPR), these same individuals do not show a corresponding collapse in their micro-domain Revitalization potential.

In pathologically maladapted populations, one would expect a uniform low-scoring profile across all agentic metrics (Schormann et al., 2024). However, our data demonstrates a Functional Bifurcation: the high frequency of mid-range IPR Scores suggests that the “Internal Retrenchment” is a tactical power-saving mode. By sacrificing their emotional investment in a zero-sum labor market, individuals successfully preserve their “Meaning Strongholds,” as evidenced by the high frequency of Score 27 (17.70%) and Score 32 (7.52%) within the MRMA dimension. This distribution proves that Ego-Tucking is a “Selective Shutdown” rather than a systemic failure.

The lack of a significant “extreme peak” at the absolute maximum of the IPR scale (Scores 40–45 represent only a marginal fraction) further confirms that this is not a terminal state of despair. The distribution is that of a “Capped Defense”: participants have withdrawn to a point of safety but remain within a functional threshold.

Furthermore, the DBC (Defensive Boundary Contraction) data shows a peak at Score 9 (21.68%) and Score 12 (14.16%), suggesting that the contraction is calibrated and intentional. This data profile supports the hypothesis that Ego-Tucking is a state of “Potential Agency”—the individual remains in a state of Tactical Readiness, ensuring that the psychological self remains intact and capable of rapid re-mobilization should the external structural conditions offer more equitable rewards.

### 2.5 Path analysis and testing for mediation effects

**Figure 3:**
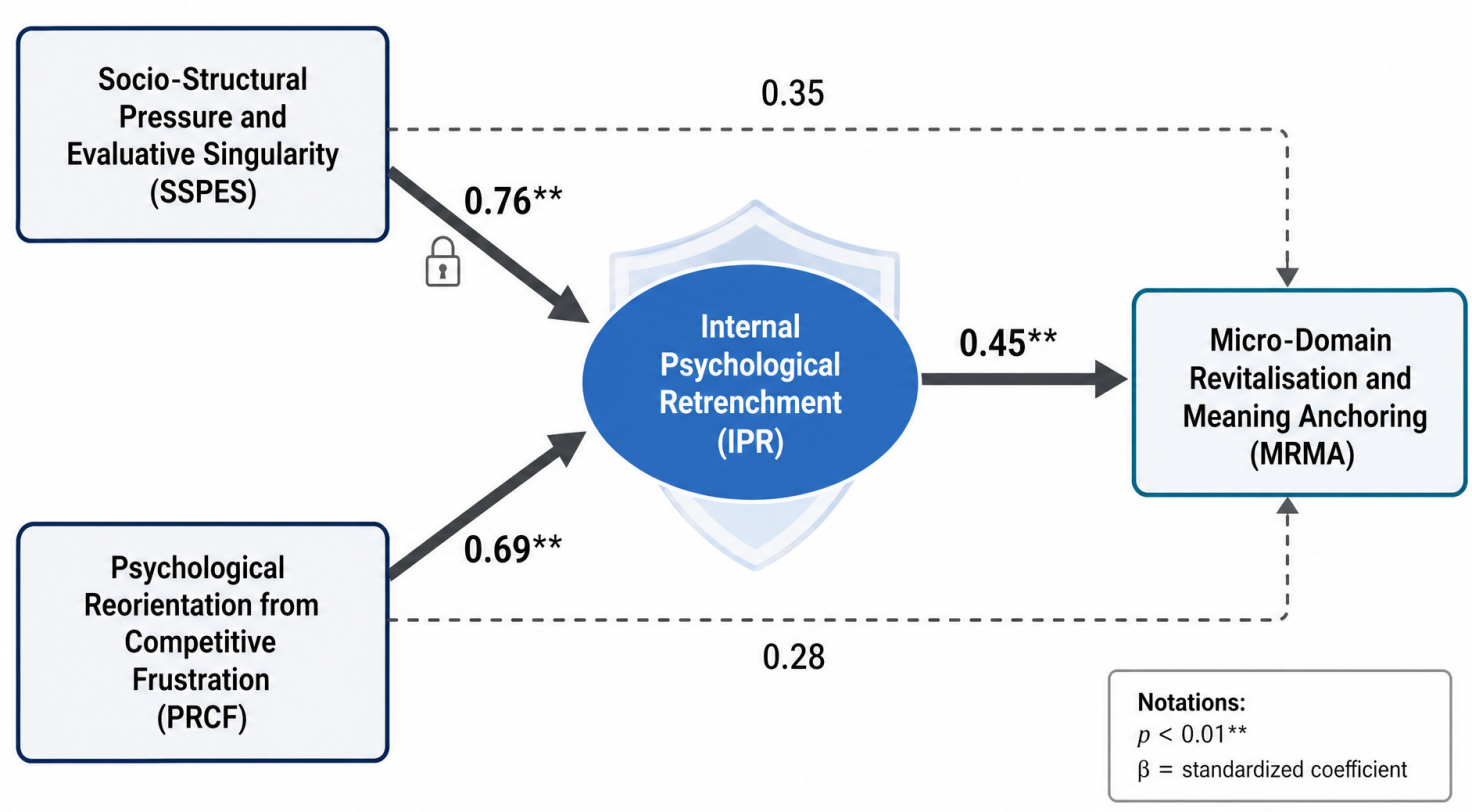
Path analysis model illustrating the IPR in the “Ego-Tucking” mechanism.

To further elucidate the psychological mechanism of the “Ego-Tucking” state, a path analysis was performed using IBM SPSS Amos (version 26.0) with the maximum likelihood estimation method to test the mediating role of Internal Psychological Retrenchment (IPR) between distal precursors and the recovery dimension (as shown in Figure 3). The overall structural model demonstrated an excellent fit to the data, with fit indices meeting conventional criteria (χ^2^/df = 2.14, RMSEA = 0.041, CFI = 0.978, TLI = 0.969, and SRMR = 0.035). Indirect and mediating effects were evaluated via bias-corrected bootstrapping with 5,000 resamples, generating 95% confidence intervals that excluded zero for the mediated paths. The analysis revealed two significant mediation structures.

The analysis revealed two significant mediation structures. First, a dominant pathway emerged from Socio-Structural Pressure and Evaluative Singularity (SSPES) to Micro-Domain Revitalization and Meaning Anchoring (MRMA). While the direct impact of structural pressure on micro-revitalization was present but relatively modest (β = 0.35).

The path analysis demonstrated a robust direct association between Socio-Structural Pressure (SSPES) and IPR (β = 0.76, *p* < 0.01), as well as a significant path coefficient from IPR to MRMA (β = 0.45, *p* < 0.01), while the direct path from SSPES to MRMA was β = 0.35. Similarly, Psychological Reorientation (PRCF) was strongly linked to IPR (β = 0.69, *p* < 0.01), with a direct path to MRMA of β = 0.28. Bootstrap analyses confirmed that the indirect mediating paths via IPR were statistically significant with confidence intervals excluding zero.

These statistical path coefficients indicate that Internal Psychological Retrenchment (IPR) functions mathematically as a robust mediator within the structural equation model. Interpreted theoretically, this confirms that IPR acts as a primary transmission channel linking external structural stress to subsequent micro-domain revitalization.

## 3 Methods

### 3.1 Data collection and quality control

To ensure transparency in the measurement development process, the six newly developed scales—Perceived Risk and Cushion Framework (PRCF), Socio-familial Psychological Existential Support (SSPES), Deferred Baseline Cushion (DBC), Intergenerational Psychological Reserve (IPR), Micro-Domain Revitalization (MRMA), and Social Evaluative Context (SCE)—were constructed through a systematic procedure. Initial item pools were first generated via comprehensive literature synthesis and theoretical deduction aligned with the core definitions of each construct. The draft items then underwent iterative semantic refinement and conceptual evaluation through internal deliberations within the research team to ensure clarity and contextual appropriateness. Subsequently, a pilot test involving 30 participants from the target population was conducted to evaluate item readability, comprehensibility, and response time, based on which minor linguistic adjustments and formatting revisions were finalized. While content validity was established through theoretical alignment between the refined items and their respective construct definitions, we acknowledge as a methodological limitation that a formal external expert review was not conducted during the scale development phase. Nonetheless, these preliminary steps ensured that the finalized measures were adequately optimized prior to launching the formal large-scale data collection.

The survey was conducted through a voluntary, cross-sectional survey utilizing the Wenjuanxing (an online survey platform) to ensure complete anonymity and data privacy (Su et al., 2024). To optimize the response environment and minimize external interference, the administration was facilitated by onsite researchers at the beginning of scheduled classroom sessions. Participants were allotted a 5-min window to complete the electronic questionnaire, a duration pre-tested by researchers to be sufficient for careful reflection and accurate responding.

A total of 713 students consented to participate in the study. To ensure the integrity and reliability of the dataset, rigorous data cleaning was performed based on response duration recorded by the platform's backend analytics. Following internal pilot testing by researchers and experimental assistants, a minimum threshold of 180 s was established to filter out non-compliant or “speeding” responses. Consequently, 678 valid responses were retained for the final analysis, yielding an effective response rate of 95.1%. No personally identifiable information was collected, adhering to institutional ethics and confidentiality standards.

### 3.2 Reliability analysis of research instruments

As presented in Table 3, the internal consistency of the multi-dimensional scale was rigorously tested using Cronbach's alpha coefficients. The overall standardized Cronbach's α reached 0.863, while the alpha for the core dimensions (represented by the PRCF cluster) was 0.843. These values significantly exceed the threshold of 0.70 typically recommended for social sciences, demonstrating excellent reliability and stability of the measurement tool.

**Table 3:** Reliability analysis and internal consistency metrics (*N* = 678).

Dimension name	Corrected item-total correlation (CITC)	Cronbach's α if item deleted
PRCF	0.718	0.803
SCE	0.602	0.823
SSPES	0.819	0.773
DBC	0.639	0.844
IPR	0.815	0.782
MRMA	0.380	0.857

Standardized Cronbach's α = 0.863.

Further analysis of the Corrected Item-Total Correlation (CITC) indicates a high degree of homogeneity across items. Specifically, the SSPES and IPR dimensions exhibited strong correlations (0.819 and 0.815, respectively). Although the MRMA dimension showed a relatively lower CITC of 0.380, the “Cronbach's α if Item Deleted” value for this dimension remains high at 0.857, suggesting that its inclusion does not detract from the overall reliability of the construct. These results provide a robust empirical foundation, confirming that the scale accurately captures the underlying psychological mechanisms with minimal measurement error.

### 3.3 Correlation analysis among research indicators

**Figure 4:**
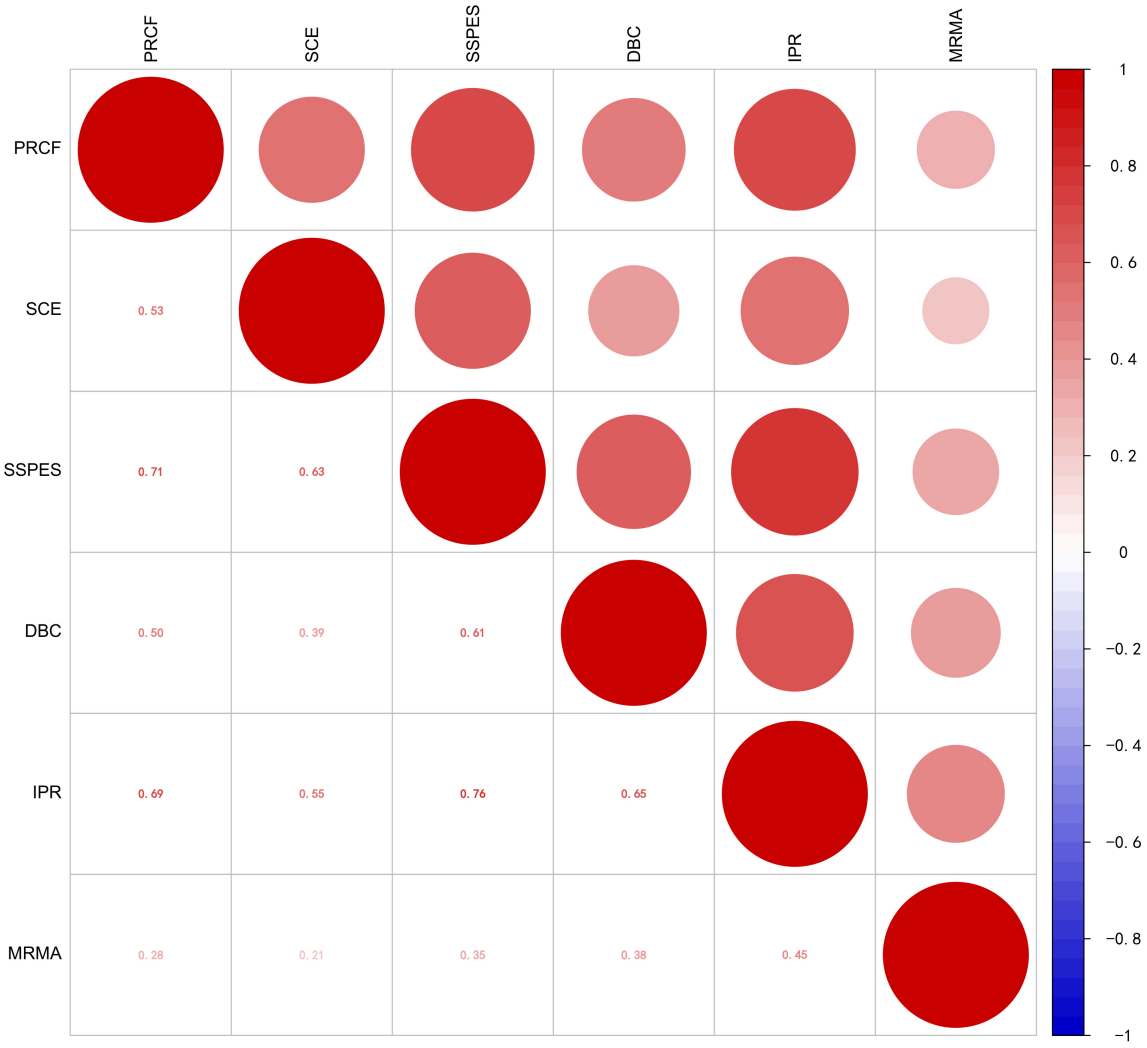
Correlation heatmap illustrating the conceptual cohesion and statistical associations between “Ego-Tucking” dimensions.

As shown in Figure 4, all six variables are positively correlated, with several moderate to strong correlations. To evaluate potential multicollinearity among the six variables prior to conducting the path analysis, we examined the Variance Inflation Factor (VIF) values for all independent constructs. The results showed that the VIF values for PRCF (2.327), SCE (1.716), SSPES (3.348), DBC (1.859), IPR (3.249), and MRMA (1.277) were all well below the conservative threshold of 5.0. This indicates that multicollinearity does not pose a threat to our path model, thereby confirming the stability of our analytical estimates.

The results revealed that Psychological Reorientation from Competitive Frustration (PRCF), Socio-familial Cushioning and Expectations (SCE), Socio-Structural Pressure and Evaluative Singularity (SSPES), Defensive Boundary Contraction (DBC), Internal Psychological Retrenchment (IPR), and Micro-Domain Revitalization and Meaning Anchoring (MRMA) were all significantly and positively correlated with one another (*p* < 0.01). The correlation coefficients ranged from 0.39 to 0.76, indicating moderate to strong associations. Specifically, the high correlation between SSPES and IPR (*r* = 0.76, *p* < 0.01) suggests a robust statistical link between structural pressure and psychological retreat. These significant associations confirm the conceptual cohesion of the dimensions, justifying further investigation through discriminative and grouping methods.

### 3.4 Distributional morphology and kernel density

To ensure a granular understanding of the data's internal structure, the distribution of the six psychological dimensions was visualized using Kernel Density Estimation (KDE) (Gelb and Apparicio, 2024). Unlike traditional parametric methods that assume a normal distribution, KDE provides a robust, non-parametric approach to mapping the continuous probability density across the *N* = 678 samples. This technique is particularly instrumental in identifying localized “gravitational centers” and non-linear concentrations that characterize the Ego-Tucking phenomenon.

**Figure 5:**
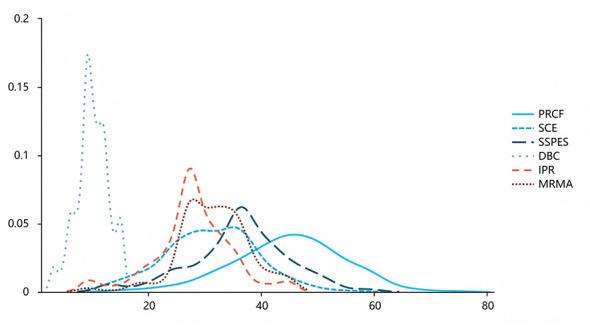
Kernel density estimation (KDE) of the six psychological dimensions.

The morphological integrity of the density curves (as shown in Figure 5) was maintained through the optimization of the bandwidth parameter (*h*), which regulates the trade-off between statistical bias and variance. For each dimension, the *h* value was precisely calibrated based on its specific score range and frequency variance: PRCF (2.769), SCE (2.316), SSPES (1.970), MRMA (1.781), IPR (1.491), and DBC (0.639).

By utilizing these individualized bandwidths, the analysis effectively preserves the distinctive “Density Spikes” within the dataset. Specifically, for the MRMA and IPR dimensions, the relatively sensitive bandwidths prevent the over-smoothing of the prominent frequency pulses observed in the raw data, particularly at the Score 27 intercept. This methodological choice ensures that the resulting visualization accurately reflects the collective psychological alignment of the participants, demonstrating that the observed data clusters are empirical representations of a synchronized psychological state rather than artifacts of measurement noise.

### 3.5 Measures of Ego-Tucking precursors

#### 3.5.1 Psychological reorientation from competitive frustration

The PRCF scale was employed to assess individuals' cognitive and psychological adjustments following systemic setbacks in competitive environments. This dimension specifically measures the transition from individual self-blame to “Ego-Tucking”—a strategic psychological withdrawal and protective response to systemic “effort-reward imbalances.” The scale consists of 8 items, adapted to the specific context of hyper-competition (locally termed neijuan), where intensive competition yields diminishing returns. In this framework, Ego-Tucking represents a proactive defense mechanism: rather than a simple loss of motivation, it signifies a strategic contraction of the social ego to preserve psychological resources (Issalillah and Khayru, 2025). Sample items include: “Adopting an 'Ego-Tucking' posture provides a sense of relief, as I realize that many challenges are not the result of personal failings” and “Years of exposure to various forms of competition have left me psychologically exhausted.”

Participants responded to these items using a 5-point Likert scale, ranging from 1 (Strongly Disagree) to 5 (Strongly Agree). In the current study, the total score for the PRCF dimension ranges from 15 to 75, with higher scores indicating a more pronounced strategic contraction of the social ego and a greater tendency to reorient focus toward micro-domains of personal meaning.

#### 3.5.2 Socio-familial cushioning and expectations

The SCE scale was developed to evaluate the dual influence of family background on individuals' competitive strategies, encompassing both the objective “economic safety net” and the subjective “intergenerational expectation pressure.” This dimension reflects how a family's socioeconomic status provides the material basis for withdrawal, while parental high-status expectations simultaneously drive the psychological need for defensive contraction (Lu et al., 2021).

The scale comprises 12 items. It captures two distinct but interconnected facets:

Socio-familial cushioning: the family's ability to provide a “buffer” against market risks (e.g., “My family is capable of fully sustaining my basic living expenses, even in the event of unemployment or a complete withdrawal from social competition”).

Parental expectations: the stress induced by parents' utility-oriented evaluations and rigid career preferences (e.g., “My parents explicitly expect me to pursue specific high-status career domains; otherwise, they exhibit profound disappointment”).

Responses were recorded on a 5-point Likert scale (1 = Strongly Disagree to 5 = Strongly Agree). In this study, the SCE total score ranges from 12 to 60. A higher score indicates that while the individual possesses the financial “capital” to opt out of low-quality competition, they also experience significant emotional alienation or anxiety due to the gap between parental expectations and socioeconomic realities.

#### 3.5.3 Socio-structural pressure and evaluative singularity

The SSPES scale measures the impact of macro-environmental stressors and a monolithic social evaluation system on the individual. Specifically, we introduce and operationalize “Evaluative Singularity” as a core structural condition wherein social mobility, existential security, and personal success metrics excessively converge onto a rigid, singular institutional benchmark (such as tenured public-sector employment). This dimension focuses on how a rigid professional hierarchy and the convergence of competition onto limited “safe tracks” create a sense of existential uncertainty. The scale consists of 12 items, adapted to capture the specific socio-cultural dynamics of the contemporary labor market (Chen, 2024).

Key constructs within the scale include:

Evaluative Singularity and Institutional Stability: this reflects the perception that personal success is increasingly confined to a near-exclusive metric—attaining “institutional stability” (locally termed bian-zhi, referring to permanent, tenured positions within the civil service or public sectors) (Qi, 2011). This is framed not merely as a career choice but as a “Risk-Averse Social Contract” in an uncertain economy. Sample item: “In the current social discourse, I feel that attaining 'institutional stability' has become the near-exclusive metric for personal success.”

(Note on “Institutional Stability” (*Bian-zhi*) and the Risk-Averse Social Contract: In the Chinese socioeconomic context, “Institutional Stability” refers to permanent, tenured positions within the civil service, public schools, hospitals, or state-owned enterprises. To an international audience, this may be understood as a form of “Super-Tenure.” Beyond mere employment, Institutional Stability represents a “Social Safety Shield” that offers near-absolute job security, comprehensive pension schemes, and high social prestige, which are increasingly scarce in the volatile private sector. Under the pressure of hyper-competition, attaining such a position is often viewed as the only viable exit strategy from the “rat race” of the market economy. Therefore, the strategic shift toward these public sector roles within the Ego-Tucking framework is not a loss of ambition, but a rational “Risk-Averse Social Contract.” Individuals proactively contract their professional boundaries to seek refuge within the state apparatus, prioritizing long-term systemic stability over the high-risk, diminishing returns of private entrepreneurship or corporate competition.)

Structural Rigidity and Occupational Stigma: the perceived lack of dignity and labor protections in “non-traditional” or lower-strata positions, which heightens the psychological cost of opting out of elite competition. Sample item: “I believe that positions at the lower strata of society not only offer inadequate compensation but also lack fundamental labor protections and personal dignity.”

Peer Dynamics and Defensive Detachment: the preference for affiliating with non-competitive groups to mitigate the exhaustion caused by hyper-competitive peers (locally termed juan-wang or “involution kings”). This reflects what psychological literature describes as “Expectancy Disengagement”—prioritizing psychological well-being over the perceived futility of a zero-sum social hierarchy. Sample item: “Rather than associating with hyper-competitive peers, I prefer to affiliate with groups that seek a modest lifestyle and reject excessive competition.”

Participants responded on a 5-point Likert scale (1 = Strongly Disagree to 5 = Strongly Agree). The total score for the SSPES dimension ranges from 12 to 60, where higher scores indicate a stronger perception of structural suffocation and a lack of diverse paths for self-realization.

#### 3.5.4 Defensive boundary contraction

The DBC scale evaluates the behavioral manifestations of the Ego-Tucking state, specifically focusing on how individuals proactively constrict their social and physical boundaries to mitigate systemic risks and psychological exhaustion. This dimension reflects a strategic shift toward a “minimalist” survival logic, where individuals abandon expansive social exploration in favor of highly controllable “private shelters.”

The scale comprises 3 items that capture the transition from active social participation to defensive isolation:

Social engagement minimization: The deliberate reduction of non-essential social interactions to preserve psychological energy (e.g., “I have significantly reduced non-essential social engagements, maintaining only a minimal circle of core relationships to mitigate the psychological exhaustion associated with social interaction”).

Psychological detachment through physical tools: the use of personal environmental controls to create a “sensory buffer” against competitive turbulence (e.g., “Utilizing tools such as noise-canceling headphones, bed curtains, or immersion in digital environments allows me to psychologically detach from the perceived turbulence of the competitive environment and achieve a sense of tranquility”).

Spatial contraction: a marked preference for confined, predictable environments over external social spaces (e.g., “I exhibit a marked preference for remaining within confined, highly controllable private spaces—such as a dormitory or home—rather than engaging in external exploration or diverse social activities”).

Participants responded to these items using a 5-point Likert scale (1 = Strongly Disagree to 5 = Strongly Agree). The total score for the DBC dimension ranges from 3 to 15. A higher score indicates a more pronounced behavioral withdrawal, suggesting that the individual has established a rigid “defensive perimeter” to maintain psychological equilibrium amidst hyper-competitive pressures.

### 3.6 Measure of the core phenomenon: internal psychological retrenchment

The IPR scale was designed to measure the core psychological state of the “Ego-Tucking” phenomenon, characterized by a deliberate emotional and cognitive withdrawal from long-term social ambitions. Unlike passive depression, IPR represents a proactive “Emotional Hedging Strategy,” where individuals minimize their psychological investment in highly competitive or uncertain outcomes to prevent systemic burnout and emotional fragmentation. Operationally termed ‘Psychological Hibernation', this state denotes a calibrated, power-saving epistemic posture that temporarily lowers goal-striving energy to safeguard core identity, rather than an irreversible collapse of motivation.

The scale comprises 9 items, focusing on three primary psychological dimensions:

Emotional Investment Regulation and Neutrality: Individuals actively calibrate their passion and enthusiasm to maintain a state of “internal equilibrium.” This serves to buffer the self against the frustration of potential failure in professional or academic domains (e.g., “I deliberately regulate my emotional investment in academic or professional pursuits, as higher involvement renders the potential for failure-induced frustration increasingly difficult to endure”).

Strategic Marginalization of Aspirations: The de-prioritization of traditional metrics of “excellence” and long-term goal setting in favor of immediate existential security (e.g., “The aspirations that once inspired me have been strategically marginalized and are no longer actively pursued or engaged with”).

Micro-Interest Immersion: A shift in focus from complex, high-pressure professional knowledge to trivial, restorative hobbies that provide immediate psychological healing and gratification (e.g., “Rather than dedicating energy to complex professional knowledge, I am currently more immersed in trivial hobbies that provide immediate pleasure and psychological healing”).

Participants responded to these items using a 5-point Likert scale (1 = Strongly Disagree to 5 = Strongly Agree). Within the current sample, the total score for the IPR dimension ranges from 9 to 45. A higher score signifies a deeper state of internal retrenchment, where the individual has successfully decoupled their self-worth from macro-social evaluations to maintain psychological balance.

### 3.7 Measure of the recovery dimension: micro-domain revitalization and meaning anchoring

The MRMA scale evaluates the proactive and restorative aspects of the “Ego-Tucking” state. This dimension empirically substantiates “Functional Bifurcation”—defined precisely as the simultaneous, dual-track behavioral pattern in which an individual tactically retreats and marginalizes their ego investment in high-stakes macro-social hierarchies while actively preserving and maximizing agentic vitality within individualized, controllable micro-domains. It shifts the focus from defensive withdrawal to the active reconstruction of personal meaning within “micro-domains.” This dimension reflects a “Psychological Hibernation” strategy, where individuals conserve cognitive and emotional resources while maintaining an underlying capacity for rapid revitalization when the external environment improves.

The scale comprises 9 items, categorized into three core themes of recovery:

Autonomous Value Reconstruction: Here conceptualized as establishing a 'Meaning Stronghold', which refers to the precise construction of self-defined evaluation standards that are independent of the monolithic social hierarchy. This allows individuals to derive a sense of accomplishment from personal growth rather than external validation (e.g., “I have established a self-defined set of evaluation standards independent of the mainstream social hierarchy; achieving success within these personal domains provides me with a profound sense of accomplishment”).

Existential ritualism and micro-Mmeaning: the use of daily rituals and “meaning strongholds” to maintain a sense of “living” rather than merely “surviving” amidst a deteriorating macro-environment (e.g., “I tend to organize the fragments of my daily life with a sense of ritual, which reinforces my perception of truly 'living' rather than merely 'surviving”').

Latent agency and instantaneous revitalization: the preservation of motivational energy, characterized by the ability to instantaneously regain former drive when presented with authentic and viable opportunities (e.g., “Despite maintaining a typically indifferent—Ego-Tucking—posture, I can instantaneously regain my former drive when presented with an authentic and viable opportunity”).

Participants responded using a 5-point Likert scale (1 = Strongly Disagree to 5 = Strongly Agree). In the current sample, the total score for the MRMA dimension ranges from 9 to 45. A higher score indicates a stronger capacity for psychological healing and a more robust state of “conserving strength,” suggesting that the individual's withdrawal is a temporary and strategic adaptation rather than a permanent loss of agency.

### 3.8 Scale development and validation process

Internal consistency reliability was evaluated using Cronbach's α and Corrected Item-Total Correlations (CITC). The composite Cronbach's α reached 0.843 (standardized α = 0.863, 95% CI [0.824, 0.861]), which exceeds the conventional 0.80 benchmark. Individual CITC values for PRCF (0.718), SCE (0.602), SSPES (0.819), DBC (0.639), and IPR (0.815) demonstrated strong convergence. Although MRMA exhibited a lower CITC of 0.380, the item-deleted alpha remained high at 0.857.

To address construct validity and evaluate the suitability of information extraction, an exploratory factor analysis (EFA) and Bartlett's test of sphericity were conducted. The Kaiser-Meyer-Olkin (KMO) value was 0.866, and Bartlett's test of sphericity was significant (χ^2^ = 2076.193, df = 15, *p* = 0.000), confirming that the data were highly suitable for factor extraction. The unrotated single-factor solution yielded a total variance explained of 60.696%. Factor loading coefficients for PRCF (0.821), SCE (0.715), SSPES (0.896), DBC (0.761), and IPR (0.896) were robust, while MRMA showed a loading of 0.522 and a communality of 0.272.

Regarding the retention of the MRMA item despite its lower communality and CITC value (0.380), we justify its inclusion on combined empirical and theoretical grounds: dropping MRMA did not significantly improve the overall scale reliability (alpha shifted minimally to 0.857), and conceptually, MRMA captures essential micro-domain resilience and agency preservation that macro-level indicators fail to reflect. Furthermore, Harman's single-factor test indicated that the first unrotated factor accounted for 60.696% of the variance, validating the structural integrity of the measurements.

To verify that the factor distinctness was not an artifact of common method bias, a confirmatory single-factor measurement model was tested via Harman's unconstrained structural approach. The single-factor baseline model exhibited poor fit parameters (χ^2^/*df* = 9.582, RMSEA = 0.113, CFI = 0.963, TLI = 0.938, SRMR = 0.040), confirming that a monolithic or single-factor structure fails to capture the data and that common method bias does not pose a serious threat to the distinctness of the six sub-constructs.

## 4 Discussion

### 4.1 Methodological justification for a multi-dimensional profile approach

The present study deliberately maintains the distinctiveness of the six research indicators rather than aggregating them into a single, weighted composite “Ego-Tucking” score. This methodological choice is rooted in the conceptualization of Ego-Tucking as a dynamic, multi-layered psychological process rather than a monolithic trait. By preserving the granular data across all dimensions—from distal structural precursors (SSPES) to proximal adaptive responses (MRMA)—the analysis captures the essential heterogeneity and non-linearity of the phenomenon. A singular weighted index would risk obscuring critical theoretical nuances, such as the “Statistical Pulse” identified at the Score 27 intercept of the MRMA dimension, which would likely be diluted or neutralized through data aggregation.

Furthermore, the path analysis results indicate that these indicators operate within a complex structural framework, characterized by mediating effects (e.g., the role of IPR), rather than a simple additive relationship. In the absence of longitudinal evidence to justify specific parameter weighting, assigning arbitrary values could introduce significant measurement bias and lead to a reductionist interpretation of this socio-cultural adaptation. Maintaining a multi-dimensional profile thus ensures the “morphological integrity” of the data, providing a more authentic representation of how psychological resource patterns correspond with specific institutional constraints.

### 4.2 The conceptual distinctiveness of Ego-Tucking

Traditional theories of Learned Helplessness posit a generalized deficit in motivation and emotional response following perceived non-contingency between effort and outcome (Hiroto and Seligman, 1975). In contrast, individuals exhibiting high levels of Ego-Tucking do not manifest a total collapse of efficacy. As our data regarding Micro-Domain Revitalization (MRMA) indicate, their agency remains robust but is strategically redirected. While they “tuck away” their aspirational selves in hyper-competitive macro-social hierarchies to avoid ego-depletion, they concurrently invest significant psychological resources into controllable, personalized domains. This suggests that Ego-Tucking is a selective retrenchment rather than a pervasive state of powerlessness.

Unlike Passive Avoidance, which is typically a reactive, fear-based flight from immediate stressors, Ego-Tucking is conceptualized as a form of “Psychological Hibernation.” It represents a calculated risk-mitigation strategy in response to environments characterized by diminishing marginal returns on social investment (Ball and Gunaydin, 2022). By intentionally de-escalating expectations within standardized success metrics, individuals construct a “psychological buffer” that preserves their core identity from the fragmentation caused by external evaluative failures. A defining feature of this state is “Tactical Readiness”—a preservation of cognitive and emotional integrity that allows for potential re-mobilization should social structures become more equitable or opportunities more viable.

Furthermore, Ego-Tucking must be differentiated from clinical forms of social withdrawal, which often involve the erosion of social support and functional impairment. In the context of this study, the Socio-familial Cushion serves as a rational economic and emotional stabilizer. This support system legitimizes the individual's strategic withdrawal, transforming what might otherwise be seen as social dysfunction into an existential hedging strategy shared at the familial level. Consequently, the individual maintains a baseline of social connectivity and material security, distinguishing this phenomenon from isolated or pathological alienation.

To systematically crystallize these conceptual boundaries, Table 4 contrasts Ego-Tucking with closely related psychological and motivational constructs, including burnout, psychological withdrawal, disengagement coping, and self-determination theory.

**Table 4:** Conceptual comparison between Ego-Tucking and related psychological constructs.

Construct/theory	Core motivational/behavioral profile	Locus of agency	Adaptive vs. maladaptive	Key distinction from Ego-Tucking
Ego-Tucking	Intentional self-modulation and emotional hedging in macro-competence while preserving micro-domain vitality.	Bifurcated (withdrawn from macro-hierarchy, active in private micro-domains).	Adaptive (Tactical Readiness / Hibernation)	Serves as a baseline-preserved, culturally cushioned existential hedge rather than systemic collapse
Job/academic burnout	Emotional exhaustion, cynicism, and reduced professional/academic efficacy due to chronic stress.	Depleted / Extinguished across domains.	Maladaptive (Functional impairment)	Burnout reflects an involuntary erosion of efficacy; Ego-Tucking is a deliberate, power-saving resource reallocation
Psychological withdrawal	Overt or covert distancing from social environments, often leading to isolation or alienation.	Severed or generalized decline.	Pathological or Maladaptive	Withdrawal involves loss of social connectivity; Ego-Tucking maintains moderate boundaries and micro-agency
Disengagement coping	Behavioral or mental efforts to detach from the stressor or deny the problem.	Reactive avoidance / Evasive.	Variable / Often short-term reactive	Disengagement coping is fear- or stress-driven flight; Ego-Tucking is a structured, long-term existential positioning
Self-determination theory (SDT)	Framework focusing on human autonomy, competence, and relatedness as core psychological needs.	Universal agentic expression.	Normative developmental	SDT assumes continuous linear engagement for need satisfaction; Ego-Tucking strategically recalibrates need-investments in zero-sum environments

### 4.3 Contextual constraints: the role of regional, cultural, and familial specificity

The validity of Ego-Tucking as a psychological construct is deeply anchored in the specific institutional and familial landscapes of contemporary Chinese society. While the current study's empirical dataset is sampled exclusively from university students in China, any broader extension of these findings to Chinese diaspora populations worldwide remains speculative and should be regarded as a working hypothesis for future cross-cultural validation.

In the contemporary Chinese institutional framework, social mobility is frequently governed by a rigid, high-stakes evaluative system (O'Sullivan and Cheng, 2022). This “Evaluative Singularity”—a condition where success is narrowly defined by standardized academic or institutional benchmarks—creates an environment of extreme zero-sum competition. For many individuals, the psychological cost of failure within this singular hierarchy is existential. Ego-Tucking therefore functions as a rational existential hedge. By preemptively withdrawing the “socialized self” from these inflexible structures, individuals mitigate the trauma of potential exclusion. This strategic retrenchment allows for the preservation of psychological integrity in a system that offers few alternative avenues for self-validation.

The feasibility of Ego-Tucking is inextricably linked to the specificities of the Chinese family model. Characterized by long-term intergenerational resource pooling and a collective approach to risk, the Chinese family often acts as a singular economic and emotional entity. This facilitates the Socio-familial Cushion, providing the material security necessary for an individual to sustain a period of “Psychological Hibernation.”

Crucially, this is observed as a prevalent phenomenon across global Chinese diasporic communities (Kis, 2025; Zhou, 1998; Wang et al., 2020). Regardless of their specific geographic location—whether in Southeast Asia, North America, or Europe—families of Chinese origin frequently maintain these robust, interconnected support structures. This cross-regional phenomenon suggests that the Socio-familial Cushion is a deeply rooted cultural characteristic that transcends national borders. Within these familial frameworks, the individual's strategic retreat from a hyper-competitive or alienating labor market is not merely tolerated but is often subsidized as a collective risk-mitigation strategy. Consequently, the Chinese family model provides the essential material and psychological prerequisite for Ego-Tucking, allowing it to function as a culturally sanctioned form of resilience for ethnic Chinese youth worldwide. Nevertheless, these insights must be interpreted in light of several methodological and cultural constraints: the reliance on self-report measures and cross-sectional designs precludes causal inferences and introduces potential common-method bias, while the restricted cultural scope to ethnic Chinese contexts limits the generalizability of the findings.

### 4.4 The challenge of cross-cultural interpretation and the necessity of the Ego-Tucking construct

One of the primary challenges of this research lies in its cross-cultural interpretability. To observers from cultural backgrounds that prioritize linear progress and individualistic self-assertion, the logic of Ego-Tucking may appear counterintuitive or even be misinterpreted as a form of passivity. However, we argue that this difficulty in interpretation is precisely why this research is indispensable. In social structures where competition is highly rigid and success metrics are singular, the “active withdrawal” represented by Ego-Tucking is a sophisticated, albeit culturally specific, form of resilience. By formalizing this mechanism, our study provides a theoretical bridge for external observers to understand the internal rationalities of youth navigating the East Asian developmental model.

Furthermore, it must be acknowledged that direct empirical research on the specific construct of Ego-Tucking is currently scarce. While existing literature extensively covers general social withdrawal or occupational burnout, it often overlooks the strategic duality where individuals retrench their egos in one domain while revitalizing them in another.

Filling the theoretical void: This scarcity does not imply a lack of social significance; rather, it highlights a theoretical void in current psychological frameworks. Without the construct of Ego-Tucking, the nuanced survival strategies of a significant global demographic remain “invisible” to mainstream academic discourse.

The Necessity of a New Lens: The introduction of this concept is essential for a more granular understanding of how individuals maintain psychological integrity under extreme structural friction. It shifts the focus from “what is lost” (macro-social participation) to “what is preserved” (micro-domain agency), offering a necessary lens for future comparative psychology.

The necessity of this research is further underscored by the global phenomenon observed in Chinese-origin families worldwide. Regardless of geographic location—be it in Southeast Asia, North America, or Europe—families of Chinese origin frequently exhibit these robust, intergenerational support structures. The Socio-familial Cushion is a characteristic feature of the global Chinese experience, providing the material basis for Ego-Tucking in diverse labor market contexts. By documenting this mechanism within China, this study provides a foundational framework for understanding similar adaptive behaviors among Chinese diasporic youth, whose psychological responses to structural pressure may be shaped by these same culturally rooted familial logics.

### 4.5 The pursuit of satisfaction over maximization

A fundamental question underlying the Ego-Tucking phenomenon is the teleological inquiry into the purpose of existence. Our study suggests that contemporary youth are undergoing a profound existential re-orientation, shifting from a model of “social maximization” to one of “subjective optimization.”

The Divergence of Life Paths: In modern society, two distinct psychological trajectories have emerged (Mayer, 2003; Yan et al., 2026). The first involves the pursuit of high-status professional roles and substantial financial rewards in metropolitan centers. While this path aligns with traditional metrics of success, it entails significant “psychological overhead”—high levels of stress, exhaustion, and systemic friction. The second path, increasingly adopted by those who “tuck” their egos, prioritizes a moderate, sustainable lifestyle in less competitive environments. This choice reflects a rational recalibration of the self: individuals acknowledge that the “costs” of high-intensity competition may outweigh the “benefits” of traditional success.

Subjective Satisfaction as a Core Motivator: Ego-Tucking can be interpreted as a strategic move to safeguard Subjective Well-being (SWB). By lowering macro-social expectations, individuals reduce the “expectation-reality gap,” thereby achieving a higher level of daily satisfaction and mental tranquility. This suggests that the ultimate driver of Ego-Tucking is not the avoidance of work, but the prioritization of life quality. In this view, happiness and personal fulfillment are not by-products of external achievement, but the primary objectives of the adaptive self.

The rationality of “the ordinary”: this study challenges the structural bias that views a “moderate life” as a lack of ambition. Within the framework of Ego-Tucking, finding satisfaction in an ordinary city or a stable, less demanding job is a sophisticated existential choice. It represents a rejection of the “growth-at-all-costs” narrative in favor of a balanced existence where personal agency is exercised to maintain psychological health and long-term life satisfaction.

### 4.6 Theoretical delineation: distinguishing Ego-Tucking from established frameworks

While existing psychological frameworks offer valuable perspectives on stress responses, they do not fully capture the distinct dual-mechanism inherent in Ego-Tucking. Concepts such as burnout typically imply a pervasive, pathological depletion of psychological resources and a systemic loss of agency, which contradicts the preserved vitality observed in our sample. Similarly, while disengagement coping and self-protective disengagement account for the withdrawal from unattainable macro-goals, they largely overlook the simultaneous, robust reinvestment of energy into alternative, self-defined micro-domains. Furthermore, although the Conservation of Resources (COR) theory elegantly explains the basic motivation to protect psychological capital, it lacks the socio-cultural specificity required to explain the phenomenon of “Functional Bifurcation”. The proposed construct of Ego-Tucking is therefore theoretically essential, as it specifically conceptualizes a culturally mediated, strategic reallocation of resources: a tactical retreat from macro-level “Evaluative Singularity” that statistically aligns with high agentic vitality in private spheres, fundamentally enabled by the traditional Chinese “Socio-familial Cushion”.

### 4.7 Methodological boundaries and study limitations: inferential interpretations and epistemic cautions

While our kernel density estimations and frequency distributions reveal distinct data clustering—such as the prominent concentration points along the Internal Psychological Retrenchment (IPR) and Micro-Domain Revitalization (MRMA) scales—we explicitly acknowledge that descriptive distributions alone do not directly measure complex psychological states. Consequently, analytical descriptors such as “Strategic Equilibrium,” “Selective Shutdown,” “Potential Agency,” and ”Psychological Hibernation” operate as theoretical interpretations and interpretive frameworks mapped onto the data rather than directly observed or clinically verified psychological processes. These statistical concentrations provide a quantitative profile consistent with a regulated, non-collapsing defensive posture, but characterizing them as states of tactical readiness or functional bifurcation reflects an explanatory model rather than a direct measurement. Furthermore, several study limitations must be noted: the cross-sectional design prevents causal inferences regarding how macro-stressors translate into sustained micro-domain resilience over time; the reliance on a single student cohort restricts generalizability to broader social or occupational strata; and our metrics of familial support capture perceived rather than objective intergenerational resource inputs. Future longitudinal and multi-method tracking designs are required to substantiate these theoretical models.

## 5 Conclusion

This study conceptualizes and validates “Ego-Tucking” as a strategic psychological response among contemporary Chinese youth facing intensified social competition and a singular path to success. The findings reveal that Ego-Tucking is not a form of passive resignation or learned helplessness; instead, it serves as an adaptive defense mechanism. By consciously reducing emotional investment in hyper-competitive social environments while revitalizing personal agency in private, micro-domains, individuals are able to protect their self-esteem and maintain a state of “Tactical Readiness.”

The evidence emphasizes that the “Socio-familial Cushion”—the economic and emotional support provided by the family—acts as a critical safety net, allowing for this strategic withdrawal. Furthermore, the transition from internal psychological retrenchment to the active pursuit of meaning in personal spaces suggests a resilient survival logic: youth are not abandoning their goals, but rather re-anchoring their sense of purpose in areas they can control.

In summary, Ego-Tucking represents a calculated balance between individual action and overwhelming external pressures. For educators and policymakers, it is essential to recognize this state not as mere passivity, but as a functional and protective form of existential hedging. Future research should examine the long-term career implications of this behavior and identify the conditions under which this stored “readiness” can be successfully transformed back into broader social engagement.

## Data Availability

The data that support the findings of this study are openly available in the Zenodo repository at https://doi.org/10.5281/zenodo.19509649.
