# Supplementary material for: Ego-Tucking: a novel psychological mechanism of strategic retrenchment and resilience among Chinese youth
Source: Front Psychol. 2026 Jul 31;17:1876249. doi: 10.3389/fpsyg.2026.1876249 (PMC13475610; doi:10.3389/fpsyg.2026.1876249)
Supplement: Supplementary file 1 [file Supplementary_file_1.pdf]

(1: Strongly disagree; 2: Disagree; 3: Uncertain; 4: Agree; 5: Strongly agree)

Psychological Reorientation from Competitive Frustration (PRCF)

1. In significant past competitions (e.g., entrance exams, proficiency tests, internships), the outcomes failed to meet my expectations despite substantial personal investment.
2. I have attempted to modify my personality or social behaviors to conform to specific institutional or social norms (e.g., interviews, student organizations), only to find such sacrifices to be of negligible value.
3. I once firmly believed that effort could alter one’s destiny, but reality has demonstrated that the returns on such efforts are significantly limited.
4. Years of exposure to various forms of competition throughout my development have left me psychologically exhausted.
5. Currently, when encountering terms like “examination,” “ranking,” or “selection,” my instinctive reaction is avoidance rather than active engagement.
6. I feel that my current psychological state lacks the residual energy required to confront further challenges.
7. Given the current labor market, individual effort is insufficient to mitigate systemic risks, such as intense competition for employment or advanced education.
8. Adopting an 'Ego-Tucking' posture—characterized by a strategic withdrawal from social competition—provides a sense of relief, as I realize that many challenges are not the result of personal failings.

(**Note on "Ego-Tucking" in the Chinese Socio-cultural Context:** The term "Ego-Tucking" is used here as a theoretical conceptualization of the Chinese phenomenon known as "Buddhist-style" (*fo-xi*) or "lying flat" (*tang-ping*). Within the current hyper-competitive environment (often described as "involution" or *neijuan*), "Ego-Tucking" represents a proactive psychological defense mechanism. Rather than a simple loss of motivation, it signifies a strategic contraction of one’s social ego to preserve psychological resources and mitigate the internal anxiety caused by systemic effort-reward imbalances. By 'tucking the ego' away from mainstream social evaluation tracks, individuals reorient their focus toward micro-domains of personal meaning.)

9. I believe the competitive pressure and frustrations I face are primarily driven by inequitable social structures and norms.
10. Observing the employment difficulties or layoffs faced by peers and relatives leads me to pre-emptively conclude that my prospects remain bleak regardless of my level of effort.
11. Frequent exposure to social media content regarding unemployment and downsizing heightens my perception of intense competition and fuels internal anxiety.
12. Social realities have compelled me to relinquish aspirations for high social status or upward social mobility.
13. Although I have lost motivation for social competition, I remain highly engaged in personal interests (e.g., romantic relationships, gaming, sports, or personal grooming).
14. My current indifferent attitude is specifically targeted at social competition; I believe my competence and talents remain intact within my chosen domains of interest.
15. I maintain a high level of engagement within online communities or interest-based digital content (e.g., commenting, liking, or following).

Socio-familial Cushioning and Expectations (SCE)

1. My family is capable of fully sustaining my basic living expenses, even in the event of unemployment or a complete withdrawal from social competition.
2. Due to my family's financial security, I do not feel compelled to accept "low-quality" employment solely for survival.
3. When facing academic or career challenges, my family’s ability to provide alternative pathways (e.g., professional connections or overseas education) serves as a significant safety net.
4. My parents have historically instilled a sense of existential crisis, suggesting that life is a constant struggle and that competition is a prerequisite for survival.
5. My parents' appraisal of me is heavily contingent upon institutional prestige, academic performance, or potential social status, rather than my personal character.
6. Since childhood, my parents have prioritized the "utility" of education and career (e.g., profitability or institutional stability) over my intrinsic interests.
7. I perceive that in my parents' view, settling for an ordinary or "low-status" job would be regarded as a personal failure.
8. My parents explicitly expect me to pursue specific high-status career domains (e.g., civil service, state-owned enterprises, or multinational corporations); otherwise, they exhibit profound disappointment.
9. To avoid acute conflict with my parents, I choose to proactively withdraw and no longer discuss my future plans with them.
10. I find it difficult to convey the severity of the current labor market to my parents, as they tend to evaluate my situation based on the "era dividends" of their own generation.
11. The career advice provided by my parents often intensifies my anxiety, as their recommendations are inconsistent with the socioeconomic realities I observe.
12. When I express stress or anxiety regarding competition, my parents often dismiss it as "fragility" or "overthinking," failing to provide genuine empathy or understanding.

Socio-Structural Pressure and Evaluative Singularity (SSPES)

1. I believe that positions at the lower strata of society not only offer inadequate compensation but also lack fundamental labor protections and personal dignity.
2. As a university student, engaging in "ordinary" manual labor would make me feel as though I have squandered years of education, making it difficult to face the scrutiny of others.
3. I perceive a rigid professional hierarchy in society, where certain occupations are widely stigmatized as "indecent" or "low-status."
4. I feel that my years of rigorous academic effort have failed to translate into the expected level of competitiveness in the current labor market.
5. I once believed that a university degree would provide an escape from the survival logic of the lower social strata, yet reality has shown that graduates face profound existential uncertainty.
6. Most available positions do not require the specialized knowledge I acquired at the university, leading to a significant sense of psychological discrepancy.
7. Rather than associating with "hyper-competitive" peers (often termed "involution kings" or *juan-wang*), I prefer to affiliate with groups that seek a modest lifestyle and reject excessive competition.

Note on "Involution Kings" (*Juan-wang*): This term refers to individuals who engage in hyper-competitive behavior within a "zero-sum" environment. In Western sociological terms, this is akin to "Status-seeking Treadmills" or "Rat Race Escalation," where individuals invest increasing amounts of effort for diminishing marginal returns. By distancing themselves from these "hyper-competitors," respondents are exhibiting what psychological literature describes as "Defensive Detachment" or "Expectancy Disengagement," prioritizing psychological well-being over the perceived futility of the social hierarchy.

8. When commiserating with peers about the futility of "involution kings" or *juan-wang* or the exhaustion of modern life, I experience a profound sense of mutual understanding and social support.
9. Observing many of my peers adopting a "Ego-Tucking" (fo-xi) or non-competitive stance makes me feel that my own state is normative and socially acceptable.
10. I find the phenomenon of everyone crowding into a few specific "tracks" (e.g., civil service exams, postgraduate entrance exams) to be suffocating due to the extreme lack of diversity in competition.
11. In the current social discourse, I feel that attaining "institutional stability" (e.g., *bian-zhi*) or high income has become the near-exclusive metric for personal success.

Note on "Institutional Stability" (*Bian-zhi*) and the Monolithic Success Metric: In the Chinese socioeconomic context, "Institutional Stability" (locally termed *bian-zhi*) refers to permanent, tenured positions within the state-owned sector, civil service, or public institutions. This preference is not merely a career choice but a strategic response to what scholars describe as a "Risk-Averse Social Contract".

12. Even if I achieve success in alternative domains such as the arts, live-streaming, or personal hobbies, those around me would likely still perceive me as having "no future.

Defensive Boundary Contraction (DBC)

1. I have significantly reduced non-essential social engagements, maintaining only a minimal circle of core relationships to mitigate the psychological exhaustion associated with social interaction.
2. Utilizing headphones, bed curtains, or immersion in digital environments allows me to psychologically detach from the perceived turbulence of the competitive environment and achieve a sense of tranquility.
3. I exhibit a marked preference for remaining within confined, highly controllable private spaces (e.g., a dormitory or home) rather than engaging in external exploration or diverse social activities.

Internal Psychological Retrenchment(IPR)

1. I deliberately regulate my emotional investment in academic or professional pursuits, as higher involvement renders the potential for failure-induced frustration increasingly difficult to endure.
2. I find it increasingly difficult to experience enthusiasm or passion for long-term objectives and future planning, maintaining a state of internal emotional neutrality.
3. In social interactions, I tend to maintain a polite yet detached demeanor to prevent the emotional fluctuations of others from disturbing my internal equilibrium.
4. The aspirations that once inspired me have been strategically marginalized and are no longer actively pursued or engaged with.
5. I have proactively relinquished the pursuit of "excellence," shifting my primary objective toward maintaining a basic level of existential security.
6. Compared to objectives requiring long-term perseverance, I exhibit a marked preference for seeking immediate achievement through daily entertainment or trivial tasks.
7. Rather than dedicating energy to complex professional knowledge, I am currently more immersed in trivial hobbies that provide immediate pleasure and psychological healing.
8. While maintaining an indifferent posture in physical social settings, I invest significant passion into online communities, virtual gaming, or specific personal interests.
9. I perceive my current lifestyle—characterized by a withdrawal from macro-goals and a focus on micro-interests—as an effective strategy for maintaining psychological balance and preventing systemic burnout.

Micro-Domain Revitalisation and Meaning Anchoring (MRMA)

1. I have established a self-defined set of evaluation standards independent of the mainstream social hierarchy; achieving success within these personal domains provides me with a profound sense of accomplishment.
2. My primary social circle has shifted toward individuals who share my intrinsic interests, creating a space that prioritizes personal passions over competitive "involution."
3. I dedicate more time to cultivating personal interests (e.g., romantic relationships, gaming, sports, media consumption, or personal grooming) as a deliberate alternative to social competition.
4. I tend to organize the fragments of my daily life with a sense of ritual, which reinforces my perception of truly "living" rather than merely "surviving."
5. Even in a deteriorating external environment, maintaining my private "meaning stronghold" provides me with the courage to confront the future.
6. I validate my existence by documenting life moments (e.g., social media posts, vlogs, or digital check-ins); this self-sufficient joy is more significant to me than external social recognition.
7. In specific moments—such as encountering evocative text, videos, or scenery—I experience a sudden restoration of hope and a resurgence of long-lost motivational energy.
8. Despite maintaining a typically indifferent ("Ego-Tucking") posture, I can instantaneously regain my former drive when presented with an authentic and viable opportunity.
9. I realize that my previous periods of "idleness" were actually a phase of conserving strength; provided the situation improves, my capacity for execution and concentration remains formidable.
